# Queer in Chem: Q&A with Dr Camille Bishop

**DOI:** 10.1038/s42004-023-00968-5

**Published:** 2023-09-30

## Abstract

*Dr Camille Bishop* is an incoming Assistant Professor in the Department of Chemical Engineering and Materials Science at Wayne State University. She obtained her PhD in chemistry at the University of Wisconsin—Madison, where she prepared glasses with liquid crystal-like packing using physical vapor deposition, after obtaining her B.S. in chemistry from the University of Chicago.

**Figure:**
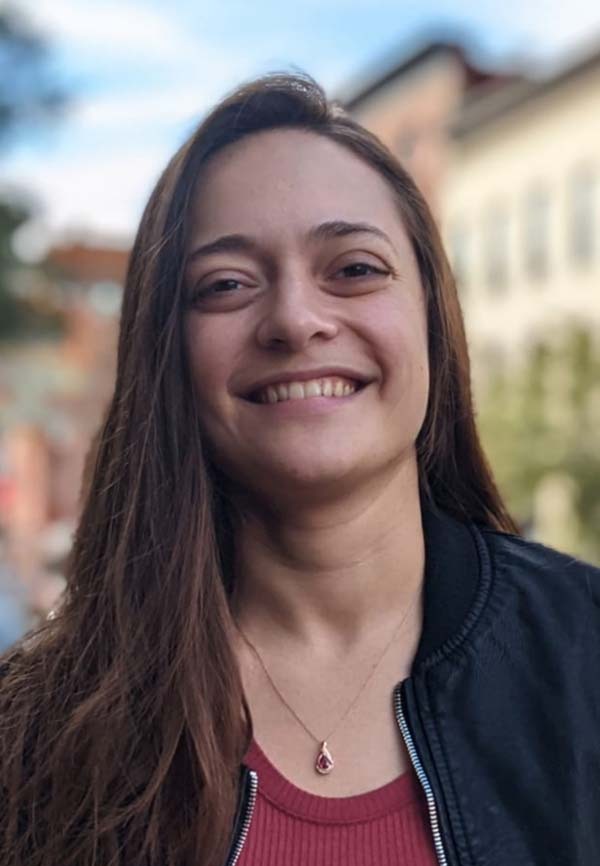
Cristina Abboud

Most recently, Camille was an NRC Postdoctoral Fellow at the National Institute of Standards and Technology (NIST), where she used resonant soft X-ray scattering to measure molecular packing in non-crystalline films. Moving into her independent career, she will investigate manipulating and measuring order in multi-component thin films with pharmaceutical and organic electronic applications. Outside of scientific research, she is passionate about outreach, learning about equity issues in science, and accumulating more hobbies than she has time for.

Why did you choose to be a scientist?

I had a really great high school chemistry teacher, and I was good at it, so I decided to major in chemistry in college. Upon getting to college, I did pretty poorly in my first-year chemistry classes, but I (incorrectly) thought that I would lose a “Women in Science” scholarship that I had if I switched majors, and I was just too stubborn. My performance improved somewhat as I moved through college, but the real switch was my first research experience.

When I started working with Paul Nealey in my undergraduate career, on a project about liquid crystal anchoring; science became more about understanding than just memorizing. I took physical and computational chemistry the same year, and suddenly everything clicked. That, and watching Paul and Juan de Pablo work together, pairing experiment and simulation, really impressed upon me how physical chemistry principles can be exploited to make real materials. A friend who was a year older than me went off to chemistry grad school and kept telling me it was the greatest thing ever. Then, Paul also encouraged me to apply to grad school, and everything just kind of escalated from there.

What scientific development are you currently most excited about?

I’m very excited about researchers using machine learning and autonomous data collection to improve the speed and quality of data collection. For example, I got to witness the development of the Autonomous Formulation Lab (AFL) at NIST, headed by my colleagues Drs. Peter Beaucage and Tyler Martin. The AFL is specifically designed to test formulations, and can be attached to many different neutron and synchrotron sources. The general approach is that the lab will make a formulation with a certain ratio of components, and then will do a scattering measurement on it to determine its phase. As it proceeds, it uses the accumulated information to intelligently guess the correct formulations to test to achieve high resolution at, say, a phase boundary, and use lower data density in a boring area. With approaches like this, researchers can optimize the time that they’re awarded at synchrotrons to obtain higher quality data, and skip measurements that aren’t useful. I think that approaches like this will really accelerate the speed at which we can answer scientific questions.

What direction do you think your field should go in?

I think that soft matter researchers need to fully lean into the computational tools that are available, which most are. I myself am an experimentalist first; however, there is no way that I can use experimental techniques to make every single one of the theoretically accessible materials that could exist. The organic small-molecule and polymer glasses that I work with are non-equilibrium materials. That means that how you prepare the film, as well as any interfaces or confinement, will change the molecular packing and the material’s function. Therefore, from a single chemical composition, you already have a wide range of possible materials. Add in different types of molecules and multi-component systems, and you have such a wide parameter space that computers and simulation really accelerate the process of intelligently selecting experimental conditions.

In the measurement field, I think approaches to data analysis that use measurement simulations will help us extract way more information from the same measurements, even without hardware improvements. Here, instead of simulating the atoms and molecules in the material, we are simulating the physics of the measurement. In my research, I use polarized resonant soft X-ray scattering (p-RSoXS). Because the X-rays have energies that are resonant with particular orbitals of molecules, you obtain a different scattering pattern at every energy. From a few scattering patterns at different energies, you can pretty quickly make some guesses about the length scales of different chemical domains in a thin film. However, I think that people often throw out too much information that is hidden in the finer details of the scattering pattern and energy dependence. To increase the information content of a measurement, we simulate scattering off of a “virtual material”, often constructed from a 2D real-space image, and compare the simulated scattering to the experimental. Through refinement of the molecular orientation fields in the virtual material, we match the scattering, giving insight into often un-measurable non-crystalline molecular orientation. This has been used, for example, to find the orientation of polymer chains in grafted nanoparticles^1^. There are similar frameworks out there, such as CREASE for neutron scattering^2^, and I think those in the field should continue efforts to develop these frameworks to extract more information from measurements.

How does your queer or trans identity intersect with your identity as a scientist?

I feel that a lot of my development as a scientist went hand-in-hand with my confidence levels surrounding my queer identity; in that way, the identities seem pretty inextricably linked. In high school, I never would have dreamed that I would be able to be publicly out. The queer people in my high school I knew were regularly bullied, so I didn’t come out to anyone. In college, I never thought that I was smart enough to be a professor one day, and didn’t reach out to others to form study groups because I thought they would think I was unintelligent. Both of those were super isolating experiences that really tested my mental health. Here I am, though. Along the way I’ve had groups of supportive people lifting me up from both the LGBTQ+ and scientific communities. Honestly, the queer groups, namely my womxn’s rugby and all-gender hockey teams, gave me the confidence boost to take chances in my education and career.

Now that I’m confident about it, sometimes I think my queer identity has actually opened more doors for me. Our society has a lot of stereotypes about how women are supposed to act and their roles in the home and professional spheres. Even the most “enlightened” of us have internalized those, myself included. Being part of the LGBTQ+ community and in a same-sex partnership, I’m constantly forced to examine and challenge those notions. In a way, I feel that some of that questioning has made me more confident in taking risks in my career.

Why do you think it is important to feel comfortable enough to bring your whole self to work?

People with traditionally respected identities have been able to bring their full selves to work since the beginning; for example, a male professor comes into work and mentions that his wife made a great dinner last night. On the other hand, a queer person may feel they have to skirt around any mention of a same-sex partner. I want all people who I work with to be able to bring their whole selves and not have it count against them; so, naturally, I need to bring my full self. So much progress in science is made through informal interactions. The more different backgrounds that are allowed to shine through, the more people can feel comfortable having the informal interactions that lead to real team chemistry.

Especially with the recent onslaught of attacks against the LGBTQ+ community in the U.S., it’s important to me for my identity to be visible. It’s easier to ignore an attack on the rights of a group of people if you don’t know any members of that group. I want to be that person sticking in my colleagues’ head to remind them that there are real people they care about who will suffer the consequences of those attacks.

How can individual scientists support and celebrate their LGBTQ+ colleagues?

I’ll start with a specific example of support that I recently received, and it confirmed that I joined the right department. The Fall meeting of the American Institute of Chemical Engineers (AIChE) this year is in Orlando, Florida, a state that is becoming increasingly dangerous for LGBTQ+ people. My new colleague reached out and asked how I would feel about him attending the meeting. After I gave him some reasoning as to why I thought that he should go, with stipulations, he settled on going and making a statement at the beginning of his talk to draw attention to the issues at the meeting.

Here are some of the ways I think colleagues could still show support while attending meetings in Florida or other states with bad civil rights records. This may also apply to other marginalized groups. The first is, do your research. Look into restaurants, cafes, and bars that are allies to the LGBTQ + (and other underrepresented) communities, and patronize those. Another is to understand that some members of marginalized groups may not be able to attend the meeting safely, and to find other recruiting and networking mechanisms so that they are not left out of the field. Finally, keep the conversation going throughout the meeting, and make sure that others are aware of the problematic location choice.

I don’t actually recommend that colleagues boycott meetings in certain states. In my view, that would only hurt the inclusion in the field as a whole. Conferences like AIChE are critical, especially for early career scientists, to meet new people and advance their career. Now for the hypothetical: If all people who are LGBTQ+ or allies boycott the meeting, the only people who will grow and advance their careers are the ones who are not allies. Additionally, it’s unfair to put the burden of the decision on LGBTQ+ scientists, who would then miss out on professional opportunities if they don’t attend the meeting. Of course, this problem can best be solved by simply not having meetings in states that are fighting against LGBTQ+ rights, although meetings must be planned several years in the future so this is not always possible. I recently read a great article that includes viewpoints from more queer and trans scientists, which I think really illustrates the nuances of the situation.

In the workplace, take a bystander intervention training. There may be other trainings on inclusivity or sensitive mentoring; those are probably also good. Bias and DEI workshops are great for people who care and want to be there; however, some studies suggest that making those mandatory can actually make the people who are already the problem even more resentful^3–5^. So, if you’re actively interested in being an ally, participate in a workshop where you can learn strategies to intervene when you see someone being harassed. Find ways you can speak up when it’s needed.

Respecting people’s pronouns and identities is a bare minimum. I know it can be hard if you’re not used to it, but it’s important to work on it. If you mess up, don’t get defensive. Apologize, correct yourself, and don’t make a big show of it.

Finally, I feel obligated to include the disclaimer that I cannot speak for all LGBTQ+ people. I still have a pretty significant amount of privilege within the community. I am white, cis, femme, and straight-passing. People don’t know that I’m queer unless I tell them, or they see me with my partner. Trans individuals, queer people of color, and very visibly queer people have different lived experiences from mine, and might disagree with the views I just expressed.

How do you lift yourself and potentially the LGBTQ+ community up to thrive in chemical research?

I think the most important strategy I use to thrive is thriving in other areas of my life that aren’t chemical research. It’s really easy to get in your head if the only value you place is on your research. That’s why it’s really important to me to be involved in outreach, keep my personal relationships strong, cook good meals for myself, and take the time to exercise and play sports.

My advice to others in the community is to remember that you’re not alone. If you’re going through a hard time with your identity and how people are treating you, know that there are people in the field who accept and celebrate you. I’m one! If you don’t know those people yet, reach out. You may try joining an LGBTQ+ affinity group in your professional society, or just reach out to someone who’s publicly out. Feeling isolated is hard, and you’ll do your best work and be happiest if you can find a group of colleagues that don’t make you feel that way. When you feel safe to, do your best to step up, live authentically, and support those who may not be able to.

*This interview was conducted by the editors of Communications Chemistry*.
